# X-linked hypophosphatemia and tumor-induced osteomalacia: a narrative review and expert opinion on the diagnostic and therapeutic challenges in the era of burosumab

**DOI:** 10.1186/s13023-025-03952-5

**Published:** 2025-10-07

**Authors:** Maria Luisa Brandi, Cristina Eller Vainicher, Danilo Fintini, Andrea Giusti, Andrea Magnolato, Salvatore Minisola, Sandro Giannini

**Affiliations:** 1 FIRMO Foundation (Fondazione Italiana Ricerca sulle Malattie dell’Osso), Firenze, Italy; 2 SC Endocrinologia, Fondazione IRCCS Cà Granda Ospedale Maggiore Policlinico, Milano, Italy; 3 Endocrinology and Diabetology Unit, Bambino Gesù Children’s Hospital, IRCCS, Rome, Italy; 4 Unit of Internal Medicine & Metabolic Bone Diseases, Villa Scassi Hospital, Regional Health Trust 3, Genoa, Italy; 5 Department of Pediatrics, Institute for Maternal and Child Health - IRCCS “Burlo Garofolo”, Trieste, Italy; 6 Department of Clinical, Internal, Anesthesiological and Cardiovascular Sciences, “Sapienza” University of Rome, Rome, Italy; 7 Dipartimento di Medicina, Clinica Medica 1, University of Padova, Padova, Italy

**Keywords:** Hypophosphatemia, XLH, TIO, FGF23, Burosumab, Diagnosis, Management

## Abstract

Hypophosphatemia presents with highly variable clinical manifestations. Among the identified hypophosphatemic disorders, X-linked hypophosphatemia (XLH) and tumor-induced osteomalacia (TIO) are caused by persistent excess fibroblast growth factor 23 (FGF23), which leads to phosphate renal wasting and reduced phosphate availability. Traditional treatments involving oral phosphate and active vitamin D supplements have limitations and potential side effects. By targeting FGF23, burosumab directly addresses the underlying pathophysiology of both XLH and TIO. This narrative review describes the diagnosis and management of XLH and TIO, highlighting key gaps and barriers within Italian clinical practice, which are often common in international healthcare settings; pragmatic solutions are also proposed to optimize patient care. Early diagnosis and appropriate treatment of XLH and TIO are crucial for preventing disease progression and improving patient outcomes. However, XLH diagnosis is often delayed or mistaken due to nonspecific symptoms, while TIO diagnosis is complicated by the challenge of localizing small FGF23-secreting tumors, which requires extensive imaging. A general lack of awareness among healthcare professionals about these rare diseases may further delay diagnosis. Management of XLH and TIO also faces hurdles. Although burosumab is now the recommended first-line treatment for XLH patients, both between 1 and 17 years old and adults, its continuous use is often limited by strict eligibility criteria, and adequate follow-up of XLH patients is difficult to maintain during the critical transition period from pediatric age to adulthood. For TIO, tumor resection remains the definitive treatment, but its success depends on tumor localization and surgical expertise. In cases where surgery is not feasible, burosumab or conventional therapy may be used, but long-term management strategies are lacking. Improving the care of XLH and TIO patients requires increased awareness, better access to advanced diagnostic tools, and enhanced multidisciplinary collaboration. Improving networking to discuss clinical cases and share best practices are crucial steps to ensure optimal patient outcomes. Implementing standardized protocols while setting personalized treatment goals and follow-up strategies can significantly improve the quality of life for patients with these rare diseases.

## Introduction

Phosphorus is an essential component for the correct functioning of multiple biological and physiological mechanisms, including plasma membrane composition, cell signaling, energy metabolism and tissue mineralization. Serum phosphorus levels are dictated by the amount of inorganic phosphate molecules ingested from the diet, their absorption into organs and tissues, and their excretion into the urine [1, 2]. Phosphate is mainly stored in bones and teeth, underscoring its crucial role in the mineralization of those tissues; smaller quantities are also present in other tissues and fluids [3, 4]. Phosphorus can also be found in organic phosphate molecules, such as phosphate esters, phospholipids, and nucleotides [2].

The primary organs that maintain phosphate homeostasis are the kidneys, bones, parathyroid glands, and gastrointestinal tract. Their interplay is facilitated by a range of tissue and circulating factors, including fibroblast growth factor 23 (FGF23), parathyroid hormone (PTH) and 1,25-dihydroxy vitamin D (1,25(OH)2D, or calcitriol). Under physiological circumstances, PTH increases renal phosphate excretion and stimulates renal synthesis of 1,25(OH)2D, which in turn increases phosphate absorption from the gut. FGF23 is predominantly secreted by osteoblasts and osteocytes and acts by reducing phosphate availability via two mechanisms: (1) it lowers renal phosphate reabsorption from the proximal tubules by downregulating the expression of sodium–phosphate cotransporters, and (2) it inhibits phosphate intestinal absorption by decreasing 1,25(OH)2D renal synthesis [2, 5]. Dysregulation of FGF23 secretion may lead to pathological phosphate imbalance [2, 6, 7].

Hypophosphatemia is a condition defined by the presence of serum phosphate levels below the lower limit of the reference range, which varies according to age [8]. In adults, serum phosphate levels below 2.5 mg/dL are classified as low [9]. In contrast, hypophosphatemia in children is defined as serum levels below 3.8 mg/dL for children aged 1–3 years, below 3.7 mg/dL for those aged 4–11 years, and below 3 mg/dL for those aged 12 years and older [8]. Clinically, hypophosphatemia is highly heterogeneous, and its manifestation and treatment options depend on the underlying etiology. Clinical presentation can range from the complete absence of signs and symptoms to the presence of skeletal deformities, muscle and/or skeletal pain, and the occurrence of stress fractures [9, 10]. As such, hypophosphatemia warrants a comprehensive differential diagnosis that should begin with a careful evaluation of the patient’s medical and family history. Various algorithms have been developed to aid clinicians in conducting a thorough differential diagnosis and preventing misdiagnoses [9–14].

To date, several hypophosphatemic disorders have been identified, arising from either reduced intestinal phosphate absorption or enhanced renal phosphate wasting. Distinct pathophysiological mechanisms can promote exaggerated urinary phosphate loss. Based on FGF23 involvement, hypophosphatemia can be broadly categorized as FGF23-independent or FGF23-dependent. FGF23-independent hypophosphatemia may arise from congenital or acquired abnormalities in the renal tubule, while FGF23-dependent hypophosphatemia results from persistent excess FGF23 [15].

X-linked hypophosphatemia (XLH) is the most common genetically inherited form of FGF23-related hypophosphatemia [12, 13], while tumor-induced osteomalacia (TIO) is one of the most frequent and well-known acquired variants. In both cases, hypophosphatemia is chronic and does not resolve spontaneously [16, 17].

Burosumab is a fully human monoclonal antibody that recognizes FGF23 and prevents its binding to the FGF receptor 1 (FGFR1)/Klotho complex, thereby neutralizing its activity. Data from the first-in-human, single-dose phase I study in adult patients with XLH were published in 2014 [18]. Based on subsequent efficacy and safety data, burosumab was approved in Europe for the treatment of children and adolescents with XLH in 2018 [19], and more recently, it received approval for treating adult XLH patients and TIO patients whose tumors cannot be localized or curatively resected [20]. As a result, burosumab has revolutionized the traditional approach to treating these diseases, and the beneficial effects observed in clinical practice highlight its potential use in a broad range of disorders characterized by FGF23 overactivity [21–23]. In Italy, burosumab has been reimbursed since March 2023 [24].

The purpose of this narrative review is to describe the diagnosis and management of XLH and TIO, highlighting the key gaps and barriers that exist within Italian clinical practice as well as in international healthcare settings, taking into account the recently updated clinical practice guidelines for XLH [14, 25, 26]. Solutions will also be proposed to assist clinicians and optimize the care of patients with these conditions.

## Methods

This manuscript is a narrative review complemented by expert suggestions, not derived through formal consensus methodology. The authors, clinicians with recognized expertise in metabolic bone disorders, reviewed key publications, including clinical guidelines, trials, and real-world evidence, regarding XLH and TIO diagnosis and management. These sources were discussed alongside their direct professional experience in managing pediatric and adult patients with XLH and TIO. Through this process, the group identified common diagnostic and therapeutic pitfalls observed in clinical practice. Based on this integrated analysis of literature and professional experience, the authors developed a set of practical suggestions aimed at improving patient care in real-world settings.

## Pathophysiology and clinical characteristics of XLH and TIO

XLH (OMIM #307800) is a rare X-linked dominant disorder affecting up to 1 in 20,000 people [27, 28] and accounting for approximately 80% of all hypophosphatemic rickets cases [12]. This disease is characterized by the presence of loss-of-function mutations in the *PHEX* gene, which results in upregulated FGF23 serum levels and consequent hypophosphatemia through the mechanisms described above [28]. XLH manifests with a wide spectrum of signs and symptoms that develop early in life (usually within the first 2 years of life), the most typical of which are limb growth retardation, abnormal walking patterns, bone pain, and rickets, which in turn result in bowing of long bones, genu varum or valgum, and skull deformities. If not promptly treated, adult XLH patients usually exhibit an exceptionally short height and may present with osteomalacia, osteoarthritis of the spine, hips and knees, enthesopathies, pseudofractures and hearing loss/tinnitus [6, 12, 29–32]. Dental anomalies and recurrent abscesses are frequently observed in XLH patients, largely due to impaired dentin and cementum mineralization [6, 33, 34]; as such, an initial dental assessment is recommended in patients ≥ 6 years old [25] and in adults [26]. In children, XLH rickets can be distinguished from rachitic lesions caused by calcium or vitamin D deficits by radiographic evaluation of the growth plates of long bones. The Rickets Severity Score (RSS) system is a valuable diagnostic tool that clinicians use to assess rickets severity and may help predict treatment outcomes. Indeed, in XLH, cortical bone is frequently thickened, and there are no indications of bone resorption [12].

Biochemical analyses can also help differentiate XLH from other causes of phosphopenic rickets. According to the latest European and international recommendations [14, 25], the panel of biochemical parameters for blood testing should include serum phosphate, calcium, alkaline phosphatase (ALP), PTH, 25-hydroxy vitamin D (25(OH)D), 1,25(OH)2D and creatinine levels. Normal or mildly elevated PTH levels help distinguish XLH from nutritional rickets, which is typically characterized by a more pronounced PTH elevation [25, 35]. Urinary calcium, phosphate and creatinine levels should also be measured to calculate the urinary calcium/creatinine ratio and the tubular maximum reabsorption of phosphate per glomerular filtration rate (TmP/GFR) [14]. The latter is particularly important for quantifying the rate of renal phosphate leakage. In addition to these biochemical parameters, serum levels of intact FGF23 should be measured. A thorough assessment of clinical, radiographic and biochemical characteristics and a detailed medical and family history are essential for XLH diagnosis, which should ultimately be confirmed by the presence of *PHEX* gene mutations assessed via genetic analysis [12, 14, 25, 26].

TIO, also known as oncogenic osteomalacia, is a paraneoplastic condition in which a tumor, mostly of mesenchymal origin (also known as phosphaturic mesenchymal tumor, or PMT), secretes excessive amounts of FGF23 [36, 37]. TIO is a rare disease described almost exclusively in adults (mean age of 40–45 years) [38, 39]; its prevalence is estimated to be 0.7 per 100,000 individuals in the overall Danish population [40], with fewer than 1000 cases being reported globally [41, 42]. Like XLH, TIO manifestations are often nonspecific and primarily related to hypophosphatemia-induced defective bone mineralization rather than the tumor itself; these manifestations include bone pain, reduced mobility, height loss, skeletal abnormalities, fractures and muscle weakness [38, 42–44]. Occasionally, the tumor can trigger local symptoms (i.e., nasal obstruction), depending on its location and size [43–45]. Along with collecting family and medical information, a physical examination should be performed to search for evident masses [46], which can be identified in more than 30% of cases, depending on the tumor size, as evidenced in the systematic review by Bosman and colleagues [41]. Similar to XLH, TIO diagnosis also requires biochemical tests, such as serum levels of phosphate, ALP, PTH, and 1,25(OH)2D and measurements of either TRP or TmP/GFR [46]. Circulating FGF23 levels should be measured after confirming hypophosphatemia, renal phosphate wasting and low or normal serum levels of 1,25(OH)2D. Although the current evidence is limited, TIO may be distinguished from XLH by the presence of higher levels of FGF23 and ALP and lower levels of phosphate and 1,25(OH)2D [47, 48]. Notably, serum FGF23 levels seem to be positively correlated with tumor size [41]. Compared with adult XLH patients, TIO patients may also have a lower bone mineral density and a greater rate of fracture [46, 47]. Compared with XLH patients [47, 48], TIO patients have also been reported to exhibit a lower incidence of osteotomy, spinal stenosis, and dental complications [47, 48]. If the test results suggest a suspected diagnosis of TIO, the location of the FGF23-secreting tumor should be determined through functional and anatomical imaging [46].

Due to the progressive and chronic nature of XLH and TIO, the correct and early diagnosis of these diseases is vital to elicit appropriate referrals and initiate adequate treatment to prevent symptom aggravation and disease progression [12, 46].

## Treatment of XLH and TIO

### Traditional approach

Before the advent of burosumab, the only medical treatment available for XLH patients consisted of oral phosphate and active vitamin D (i.e., calcitriol or alfacalcidol), commonly referred to as “conventional therapy.” However, recent European guidelines discourage using the term “conventional,” as most pediatric patients with XLH now receive burosumab [14]. Currently, treatment with oral phosphate and active vitamin D should be reserved for cases where burosumab is unavailable or the patient is ineligible for treatment [14]. In children, the recommended initial daily dose of phosphate salts (calculated based on elemental phosphorus) ranges between 20 and 60 mg/kg body weight, which should be divided into six daily doses and adjusted based on age and symptom severity [12, 14, 49]. The recommended daily dose of calcitriol is 20–30 ng/kg body weight divided into one to three administrations; alternatively, alfacalcidol can be administered once daily at a dose of 30–50 ng/kg body weight. Regardless of body weight, calcitriol and alfacalcidol can also be started at daily doses of 0.5 µg and 1 µg, respectively, in children older than 12 months [12, 14, 29, 49]. In general, greater doses are needed during the growing phase, and adjustments are required based on the serum levels of ALP and PTH, urinary calcium excretion and side effects [12, 29]. In the medical literature, a wide variety of calcitriol and elemental phosphorus doses are used in XLH patients, highlighting the absence of an ideal dose as well as concerns about the potential side effects of the therapy (discussed below) [29]. Treatment with phosphate and active vitamin D supplements is recommended in adults with XLH to reduce osteomalacia and its manifestations, employing lower doses of phosphate salts and calcitriol than those used in children (daily doses of 750–1600 mg of phosphate salts and 0.50–0.75 µg of calcitriol or 0.75–1.5 µg of alfacalcidol). Doses should be lowered in the long term to minimize the risk of complications [26]. Treatment is recommended only if patients have symptoms, including bone and muscle pain, pseudofractures, and dental problems, whereas treatment should be avoided in asymptomatic XLH patients [12, 14]. In the presence of mild symptoms, treatment with active vitamin D without phosphate should be administered [26]. If patients show persisting or worsened limb deformities, corrective surgery with either guided growth techniques or osteotomy should be considered to improve growth and prevent the development of osteoarthritis and abnormal mobility [14, 50–52]. However, complications from the procedure may arise, and recurrence cannot be excluded [53].

Complete resection of the tumor is the only definitive cure for patients with TIO, as it rapidly restores biochemical parameters within the normal range. For this reason, surgical removal of the tumor represents the first-line therapy for this disease [42, 54–56]. However, locating and removing the tumor may be unfeasible, as the tumor could be small and positioned in parts of the body that cannot be resected because of the elevated risk of complications/tissue injuries [45, 56]. In addition, a substantial portion of patients may present persistent TIO or relapsed disease even after tumor excision. Few studies have evaluated the rate of refractory or recurrent disease after surgery; among 230 patients, Li et al. reported that 18.3% of patients had either recurrent or refractory tumors after a median time of 33 months post-surgery [57]. In contrast, Bosman and colleagues reported a recurrence rate of 16.1% in a systematic review involving 895 TIO patients [41]. If the tumor is inoperable or can be partially removed, an alternative option to surgery is image-guided tumor ablation, a minimally invasive procedure that can destroy the tumor *in loco* through different means (i.e., temperature, radiofrequency) [58–60]. Although evidence is still limited, radiotherapy may also be successful in either partially controlling the disease or leading to complete TIO remission [61].

Medical treatment should be initiated for both patients who are undergoing surgery and patients with unresectable tumors [46]. The goal of preoperative medical treatment is to prepare bones for surgical procedures and prevent “hungry bone syndrome” (HBS), a condition characterized by hypocalcemia and secondary hyperparathyroidism in which bones are being remineralized, often observed after TIO excision [62]). In inoperable patients, medical treatment aims to reduce symptoms and normalize biochemical levels. Among medical treatments, therapy with oral phosphate salts and active vitamin D supplements is the longest-established approach and is particularly indicated for patients undergoing surgery [46]. According to the latest consensus, the therapy should be administered multiple times per day to reach the recommended daily doses of phosphate salts and calcitriol, which are 20–40 mg/kg and 20–30 ng/kg, respectively (alphacalcidol can replace calcitriol and be administered at a dose 1.5–2-times greater than that of calcitriol) [55]. Alternative therapeutic strategies, including the use of octreotide (a somatostatin analog that binds to somatostatin receptors [SSTRs], which are highly expressed by PMTs), have been assessed, but the results are controversial [63–65].

Oral phosphate and active vitamin D therapy can improve XLH and TIO symptoms and prevent the development of related complications. Nevertheless, it is associated with many disadvantages. First, it is a replacement therapy, and as such, it does not correct the pathophysiological mechanism leading to hypophosphatemia [29, 34, 45]. Consequently, phosphate salts and vitamin D supplements fail to restore phosphate homeostasis and normalize serum phosphate levels. In XLH patients, therapy with oral phosphate and active vitamin D does not consistently prevent the progression of the disease, with variable efficacy observed in correcting biochemical and radiological features and reducing dental problems and enthesopathy [66, 67]; paradoxically, it can further aggravate the condition by increasing FGF23 levels [68].

Therapy with oral phosphate and active vitamin D may cause or exacerbate serious complications such as nephrocalcinosis, hypercalcemia and secondary/tertiary hyperparathyroidism, which warrant additional treatments (i.e., cinacalcet, parathyroid surgery) [12, 45, 46]. This is why treated patients should be strictly monitored, and the doses should be titrated accordingly. The frequent daily administration and gastrointestinal side effects of the treatment (e.g., nausea, diarrhea, abdominal pain) substantially compromise patient compliance [69, 70].

### Burosumab

As mentioned above, burosumab represents a valid alternative to therapy with oral phosphate and active vitamin D because it offers many advantages. In contrast to therapy with oral phosphate and active vitamin D, the antibody works by blocking the action of FGF23, thereby directly addressing the primary cause underlying both XLH and TIO. Robust data from RCTs have demonstrated the efficacy of burosumab in children [67, 71–79] and adults [80–86] with XLH and in patients with TIO [87–89]. The efficacy of burosumab was demonstrated in the pivotal 64-week-long phase III UX023-CL301 study. Burosumab was superior to therapy with oral phosphate and active vitamin D in improving the severity of rickets, increasing and maintaining the serum phosphorus, ALP, and 1,25(OH)2D levels within the normal range, and increasing the TmP/GFR in children aged 1–12 years who previously received conventional therapy [72]. Later, the efficacy of burosumab and conventional therapy was compared between young (1 to < 5 years) and older (5–12 years) children; the results confirmed that burosumab improved rickets and lower limb deformities to a greater extent than conventional therapy in both age categories [67]. Recent data from the phase III extension study confirmed the amelioration of phosphate metabolism and rickets at up to 88 weeks, both in children who continued burosumab treatment and in children who switched from therapy with oral phosphate and active vitamin D to burosumab. No new safety signals were observed [79].

The efficacy of burosumab was also confirmed in symptomatic adults with XLH, showing improvements in stiffness, fracture healing, several osteomalacia-related outcomes, serum phosphorus levels (measured at midpoints of dosing intervals) and biomarkers of bone formation and resorption [80, 81]. Importantly, the efficacy of burosumab was sustained over time; the treatment was well tolerated and not associated with new safety signals during the long-term observation period (up to 184 weeks) [82]. A post hoc analysis demonstrated that burosumab was consistently superior to placebo in 14 clinically relevant subgroups of adult patients with XLH after 24 weeks of treatment, suggesting the broad efficacy of the biologic regardless of the patient’s demographic, clinical and functional characteristics [85].

Two pivotal RCTs have illustrated the efficacy of burosumab in patients with TIO. In both trials, patients achieved serum phosphorus levels above the lower limit of the normal range (up to 112 and 144 weeks) [87, 88]; in parallel, several osteomalacia-related parameters significantly improved (reduction in osteoid volume/bone volume, osteoid thickness, and mineralization lag time at week 48) [88]. Up to 33% of fractures and active pseudofractures were completely healed, and patients reported less pain and fatigue and increased physical functioning [87, 88]. The effects of burosumab are also being evaluated in real life; long-term retrospective studies (ranging from 1 to 3.3 years) assessing the effectiveness of burosumab in children and adolescents with XLH confirmed the rapid and sustained improvement in rickets and biochemical parameters [90–92], even if mild hypophosphatemia persisted in some patients [91, 93]. In a German prospective study, treatment with burosumab in pediatric XLH patients was associated with improved health-related quality of life (HRQoL) [94]. The beneficial effects of burosumab on clinical outcomes and HRQoL were also confirmed in a longitudinal study involving a cohort of 143 patients (from children to adults, median age: 17.5 years) [95]. Interestingly, burosumab was found to improve height in a cohort of 19 Brazilian adults with XLH [96]. Although there are no observational studies of burosumab in TIO patients, various case reports have indicated that increased serum phosphate levels decrease pain and improve physical function [97–99] and fracture healing [97] in TIO patients who were not eligible for surgery [97, 99] or whose tumors were not completely excised [98].

Owing to the superior efficacy and safety of burosumab over therapy with oral phosphate and active vitamin D [72] and the beneficial effects demonstrated in real life [91–95], burosumab is now the recommended first-line treatment for children and adolescents with XLH aged 1–17 years [14]. International guidelines recommend the use of burosumab in adult patients with fractures or pseudofractures in the absence of prior therapy and further suggest its preferential use over oral phosphate and vitamin D therapy [26]. Recently, the criteria for determining a satisfactory response to burosumab therapy in XLH children and adults have also been outlined. In children, a satisfactory response is characterized by significant improvements in renal phosphate wasting, serum phosphate levels, and rickets activity (including bone pain and ALP levels) within the first six months of treatment. By 24 months, sustained improvements in leg deformities, normalization of ALP values, and a growth velocity exceeding the 25th percentile for sex and age are expected. In adults, a satisfactory response is defined by significant improvements in renal phosphate wasting, serum phosphate levels, and musculoskeletal pain within six months, with further improvements in musculoskeletal pain, stiffness, and osteomalacia-related radiological lesions within 12 months. Importantly, the response in terms of growth is more pronounced when burosumab treatment is initiated early in life [14]. Burosumab is administered via subcutaneous injections; the initial recommended dose (ranging between 0.3 and 1.0 mg/kg) and administration frequency (either 2–4 weeks) vary according to the disease and patient’s age. Dose adjustments are allowed according to serum phosphate levels in both XLH and TIO patients (up to 2.0 mg/kg body weight for a maximum dose of either 90 mg or 180 mg) [20]. While complete tumor excision (if feasible) remains the primary treatment option in patients with TIO, burosumab is generally preferred to conventional therapy because of its efficacy, safety and dosage, especially in patients with recurrent or non-operable tumors [100].

## Challenges in the diagnosis and management of XLH and TIO

As highlighted above, many aspects should be carefully considered to ensure accurate diagnosis and effective management of patients with XLH or TIO. Although various international consensus, evidence-based recommendations and guidelines have been published to assist clinicians in their daily practice [12, 14, 25, 45, 46, 55, 101–104], many unmet needs persist, perpetuating diagnostic delays and suboptimal patient care. In the following sections, we delve into the major challenges associated with diagnosing and managing patients affected by these rare conditions.

### Barriers to early diagnosis of XLH and TIO

The diagnosis of XLH and TIO presents significant difficulties, often resulting in missed diagnosis and/or delayed recognition and treatment initiation, thereby accelerating disease progression. Here, we summarize the shared and specific obstacles preventing the timely diagnosis of XLH and TIO.

1. *Nonspecific signs and symptoms*: Both XLH and TIO manifest with a wide array of signs and symptoms that are often mild and nonspecific and could lead to misdiagnosis. In XLH, symptoms typically evolve with advancing age, and patients may erroneously receive diagnoses of nutritional rickets, ankylosing spondylitis and diffuse idiopathic skeletal hyperostosis [105–107]. TIO has also been mistaken for various disorders, including intervertebral disc herniation, spinal arthritis (ankylosing spondylitis), osteoporosis, hereditary hypophosphatemia (including XLH), rheumatoid/inflammatory arthritis, partial Fanconi syndrome and other bone disorders such as Paget’s disease [45, 47, 108–110]. TIO can also be misdiagnosed as primary hyperparathyroidism, as PTH levels are sometimes elevated [46]. The overall rate of misdiagnosis is estimated to be as high as 95% [108], and the amount of time between TIO initial symptoms and diagnosis/treatment spans between 0.1 and 42 years [41].
2. *Tumor localization in TIO patients*. The TIO diagnostic approach and treatment decision can be further compromised by the difficulty in localizing the FGF23-secreting tumor within the body.
3. *Awareness and expertise in XLH and TIO*. Given the rarity of XLH and TIO, there is a general lack of awareness surrounding these disorders among healthcare professionals (HCPs), and specialized expertise in XLH and TIO is mostly confined to a few specialized centers. Beyond these centers, clinicians’ awareness of these rare diseases is often scarce. It precludes the establishment of multidisciplinary collaboration, which is essential for a comprehensive assessment of the patient and the correct identification of the disease [12, 45, 46, 55].
4. *Serum phosphate measurement*. The measurement of serum phosphate levels determines hypophosphatemia; however, this parameter is not always included in routine blood tests [42]. According to recent guidelines, serum phosphate levels can be measured under non-fasting conditions. However, clinicians should take into account the interval between the blood draw and the last meal; in uncertain cases, repeated measurements are advised [14]. In addition, interpretation should account for age-specific reference ranges. In children with XLH, levels are normal after birth and usually decrease not earlier than 3–6 months of age [111].
5. *Serum ALP measurement.* Elevated serum ALP levels are a hallmark of all types of rickets [112]. Both total ALP and bone-specific ALP can be measured to assess bone turnover. In the context of normal liver function, bone-specific ALP accounts for approximately 90% of total ALP activity in children and about 50% in adults. For this reason, bone-specific ALP is preferred for monitoring disease activity in adult patients with XLH, but its use remains infrequent [113].
6. *Renal phosphate wasting quantification*. The quantification of renal phosphate wasting by TmP/GFR calculation is recommended for the diagnostic workup of both XLH and TIO [14, 25, 46]. TmP/GFR is generally preferred to tubular reabsorption of phosphate (TRP), as the latter may vary according to phosphate intake through the diet and may not account for the serum phosphate circadian variation [114]. Nevertheless, laboratory measurements of the TmP/GFR are not widely available. In addition, the test is cumbersome for patients because it involves fasting blood and urine collection. However, in children, fasting is not strictly required, and the calcium-to-creatinine ratio can be calculated from a random spot urine sample [25].
7. *Genetic testing*. The lack of genetic analysis of the *PHEX* gene impedes the definitive confirmation of XLH diagnosis, potentially undermining the accuracy of XLH epidemiological studies. As exemplified by the analysis conducted by Hawley and colleagues, the absence of *PHEX* genetic confirmation leads to the identification of individuals that are only potentially, rather than definitively, affected by XLH [115]. Even when genetic tests are conducted, results may sometimes be inconclusive, especially if they reveal a *de novo* variant of uncertain significance (VUS). While research-based tools exist to assess the pathogenicity of VUS, they are often difficult to access [25]. Importantly, if no pathogenic variants are identified in the *PHEX* gene, additional genes associated with hereditary hypophosphatemia, such as *FGF23*, *DMP1*, and *ENPP1*, should be analyzed to support an accurate diagnosis [14]. While next-generation sequencing (NGS) enables the simultaneous analysis of multiple genes and is well suited for detecting point mutations, small insertions/deletions, and splice site alterations, it may fail to identify larger structural variants. Therefore, NGS should be complemented by multiplex ligation-dependent probe amplification (MLPA), which is specifically designed to detect large exon-level deletions or duplications within single genes. The combination of NGS and MLPA provides a complete genetic assessment, enhancing diagnostic accuracy and avoiding false negatives. However, access to these tests is limited because of the high costs and/or the lack of dedicated professionals and required equipment.
8. *FGF23 quantification.* In the absence of molecular genetic analysis, a diagnosis of XLH is supported by a family history consistent with X-linked inheritance and elevated intact FGF23 levels in the presence of hypophosphatemia [14]. To date, various FGF23 assays are commercially available for detecting either intact or cleaved C-terminal fragments of the protein. Two studies identified assay-specific thresholds for intact FGF23 to differentiate FGF23-mediated from non-FGF23-related hypophosphatemia: a 27-pg/ml cut-off using the Immutopics ELISA [116] and a 30-pg/ml cut-off using the Kainos ELISA [38]. Nevertheless, results can be difficult to interpret as they vary depending on the test used [14]. In addition, FGF23 levels are influenced by age and sex [117].
9. *Imaging investigations.* Initial radiological assessment of long bones is crucial for evaluating children with suspected XLH [25]. Characteristic radiographic features (i.e., widening, cupping, and fraying of the metaphysis and sclerosis of the distal ends) are typically more evident in younger children [112]. As children age and growth velocity decreases, these signs tend to become less pronounced. This age-dependent variability can make the radiographic evaluation of rickets more challenging in adolescents. In adults, active rickets no longer occur due to the closure of growth plates. Instead, pseudofractures (Looser zones) may be present, although these are often difficult to detect on conventional radiographs, particularly in early or mild cases. In cases of suspected TIO, PMTs are usually small and can develop anywhere in bones and soft tissues. For this reason, the entire body must be scanned multiple times using functional and anatomical imaging techniques, causing significant radiation exposure [45].

**Fig. 1:**
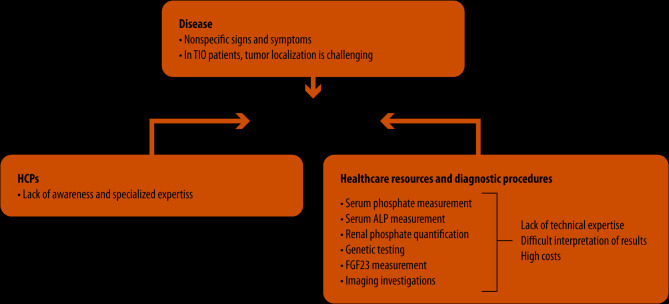
Disease, healthcare professionals and resource-related barriers to the early diagnosis of X-linked hypophosphatemia and tumor-induced osteomalacia in clinical practice HCP: healthcare professional; TIO: tumor-induced osteomalacia; XLH: X-linked hypophosphatemia

Collectively, these challenges that interfere with the XLH and TIO diagnostic pathways underscore the pressing need for increased HCP awareness and global expertise, as well as facilitated access to multidisciplinary teams and advanced technologies. An exhaustive assessment of the patient’s medical history and clinical manifestations is mandatory to guide the diagnostic process, accelerate treatment and mitigate disease progression. Figure 1 provides an overview of the difficulties in diagnosing XLH and TIO, categorized as diseases, HCPs, and resource-related challenges.

### Challenges in the management of XLH and TIO

In this section, the key challenges in managing patients with XLH or TIO in routine clinical practice are described.

**Fig. 2:**
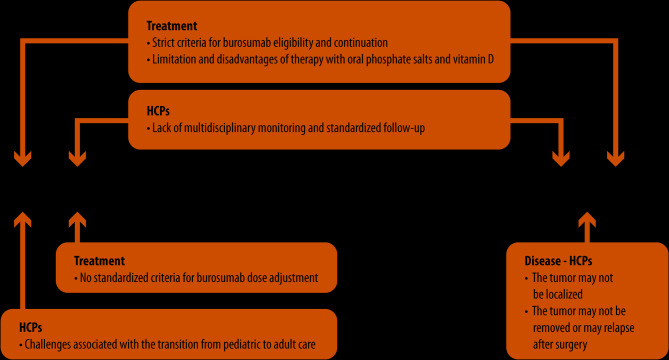
Disease-, treatment- and healthcare professional-related challenges in the optimal management of X-linked hypophosphatemia and tumor-induced osteomalacia patients HCP: healthcare professional; TIO: tumor-induced osteomalacia; XLH: X-linked hypophosphatemia

1. *Eligibility for burosumab.* According to the latest guidelines, burosumab has replaced therapy with oral phosphate and active vitamin D as the first-line treatment for XLH patients between 1 and 17 years old and is also recommended in adult patients with fractures or pseudofractures [14, 26]. Medical treatment with either burosumab or therapy with oral phosphate and active vitamin D is recommended for eligible TIO patients who cannot undergo curative surgery or who relapse [46, 55].
  In XLH patients, there are specific eligibility criteria to be met for burosumab use, reimbursement and continuation, which differ across European countries and limit its access to patients to various extents. In Italy and other countries (Germany and the UK), burosumab can be prescribed for all children aged > 1 year in whom XLH has been diagnosed either by genetic testing or with FGF23 serum levels > 30 pg/mL; these children have a growing skeleton, and bone disease is radiographically confirmed (RSS > 1.5) [101]. According to these regulations, all children diagnosed with XLH before 1 year of age can only receive therapy with oral phosphate and active vitamin D, which comes with several limitations and disadvantages that may be further exacerbated due to the young age of the patients. Additionally, according to current reimbursement conditions, burosumab should be discontinued once skeletal growth terminates. However, XLH is a chronic and progressive disease whose symptoms are likely to reappear during adulthood. Although evidence guiding the optimal therapeutic approach from childhood to adulthood is limited [25], burosumab treatment should be continued without interruption beyond the completion of longitudinal growth, at least until peak bone mass is achieved (usually during young adulthood), to maximize bone mass accrual [14]. Accordingly, two case series have shown that interrupting burosumab therapy in adolescents and young adults led to a reversal of all previously observed improvements, negatively affecting several biochemical and clinical outcomes and worsening quality of life [118, 119]. While in patients between 1 and 17 years old, the recommended initial dose is 0.8 mg/kg of body weight every 2 weeks, the starting dose in adults is 1.0 mg/kg of body weight every 4 weeks [20]. However, the ideal timing for transitioning from the pediatric biweekly dosage to the adult monthly regimen has yet to be determined [14, 25].
  In Italy, reimbursement for burosumab can be maintained in children and adolescents if its effectiveness is demonstrated by improvement in the RSS 12 months after burosumab initiation. However, such a result may not be associated with an appreciable amelioration of bone deformities. Similar criteria for the continuation of burosumab treatment have been established in other countries [101, 113]. Notably, burosumab reimbursement criteria are more stringent for adults than for children; among these, the most critical is the presence of at least one active fracture or pseudofracture. In fact, a child who has previously been treated with burosumab will probably be free of fractures and pseudofractures when reaching adult age. Hence, burosumab treatment would have to be interrupted according to current prescription requirements.
2. *Pediatric to adult care transition.* The hurdles outlined above in maintaining continuous treatment with burosumab further exacerbate the difficulties in ensuring optimal care during the growth of XLH patients, particularly during the transition from childhood through adulthood. This is indeed a critical period wherein adolescent XLH patients may experience a greater disease burden due to an evolution in musculoskeletal symptoms as well as an increase in psychological consequences while facing a new healthcare setting and interacting with different HCPs. Addressing the peculiar challenges of this period is pivotal to ensure that patients are not lost to follow-up and that they adhere to the treatment, preventing disease progression and/or complications [120–122].
3. *Burosumab dose adjustments in patients with XLH*. Due to the physiological and phenotypical changes associated with XLH, burosumab dose should be carefully titrated during patient growth to maintain clinical benefit [25, 101]. Notably, FGF23 assays cannot discriminate between free FGF23 and FGF23 bound to burosumab, meaning that treated patients may show falsely high serum levels of FGF23 [123]; therefore, this parameter should not be monitored in burosumab-treated patients. Response to burosumab has been defined in recent guidelines and requires periodic evaluation of clinical and biochemical parameters. However, no definitive cut-off values have been established for expected changes, as the extent of improvements largely depends on the baseline severity of the disease [14]. As a result, decision-making regarding burosumab dose modifications remains challenging due to the lack of standard criteria.
4. *Tumor removal.* The management of TIO patients also faces specific challenges. Complete resection of the tumor is the only definite treatment; however, the tumor may not be removable. Even if the surgery is feasible, the success of the procedure is not guaranteed because it strongly depends on the position of the tumor and the experience of the surgeon [45]. Although rare, tumor recurrence or metastasis may occur because of incomplete resection and/or the persistence of tumor cells. Image-guided ablation of the tumor can be performed instead of surgery; positive outcomes have been observed after TIO radiofrequency ablation [58]. However, experience in TIO ablation is still limited and may not be effective in all patients [124], and the long-term outcomes are still unknown [45].
5. *Multidisciplinary monitoring*. The need for multidisciplinary management of XLH patients throughout their entire lifetime is well recognized and has been widely emphasized [12, 25, 104, 120, 121, 125]. Recent guidelines highlight the importance of involving bone specialists to ensure orthopedic and rehabilitation interventions that can alleviate musculoskeletal pain and enhance physical function in those with persistent symptoms [14]. Nevertheless, achieving active and standardized collaboration among multiple specialists who should be consistently involved in patient care across various life stages remains challenging in clinical practice. The lack of age-specific multidisciplinary strategies represents a clinically unmet need and further increases the burden perceived by XLH patients.
  To date, there are no standardized protocols to ensure rigorous follow-up of TIO patients. Due to the (low) risk of tumor recurrence and the risk of developing HBS (immediately after surgery), clinicians should frequently monitor biochemical parameters even if successful tumor resection has occurred. The formation of multidisciplinary teams is crucial for the appropriate care of both patients who have undergone surgery and those receiving only medical therapy. However, there are no specific follow-up guidelines, and implementing a multidisciplinary approach may be unachievable due to the rarity of the disease and the scarcity of expert specialists.
6. *Therapy with oral phosphate and active vitamin D*. If patients with XLH or TIO are not eligible for burosumab, therapy with oral phosphate and active vitamin D is the only therapeutic alternative for managing the disease. However, this approach is considered inadequate because of the several limitations and risks discussed above. Moreover, therapy with oral phosphate and active vitamin D is not always reimbursed, and the poor palatability of galenic formulations can negatively affect young patients’ adherence [126]. The specific and shared challenges for the management of XLH and TIO are outlined in Fig. 2.

## Expert opinion

### General considerations

Given the rarity of XLH and TIO, clinical expertise is restricted to a handful of centers within each country. Active networking between the most experienced clinicians and HCPs potentially involved in the diagnosis and/or management of these rare diseases (including pediatricians, internists, endocrinologists, orthopedic consultants, radiologists, rheumatologists, physiotherapists, dentists, oncologists, psychologists) is warranted to increase the general awareness about XLH and TIO pathophysiology, their clinical consequences, and how to address them according to the patient’s age. Therefore, we strongly encourage the sharing of clinical cases that exemplify successful diagnosis and management, serving as valuable examples and promoting the implementation of best practices in patient care. Such cases serve as valuable examples, facilitating knowledge exchange, fostering professional networking, and raising awareness of recent updates in XLH diagnosis and management to enhance their implementation in patient care.

The multidisciplinary approach to diagnosing and treating these diseases has been broadly emphasized. Bone specialists and experts in metabolic bone diseases should always be present in the multidisciplinary team, as their expertise is essential for managing musculoskeletal symptoms and enhancing physical function [12, 14, 26]. Below and in Table 1, we report some suggestions for improving the diagnosis and management of XLH and TIO in clinical practice. Most of the advice provided can be applied to both conditions, while certain suggestions are disease-specific.

### Suggestions for improved diagnosis of XLH and TIO

Measurements of serum and urine phosphate, which are easily accessible and widely performed, are fundamental for diagnosing XLH and TIO; therefore, these tests should be the first to be promptly requested in cases of suspected XLH or TIO. If hypophosphatemia is detected, the TmP/GFR should be subsequently evaluated. The TmP/GFR can be calculated using the Walton & Bijvoet nomogram [127] or via online platforms. Importantly, in pediatric patients, fasting is not strictly required at the time of testing [14], as the Brodehl et al. formula allows for reliable TmP/GFR under both fasting and non-fasting conditions [128]. Furthermore, 24-hour urine collection can be avoided; a random spot urine sample is considered suitable for calculating calcium excretion in this patient setting [14, 25].

Additional methodological considerations should be noted to improve diagnostic accuracy. First, blood and urine samples should be taken simultaneously to measure phosphate and creatinine, as TmP/GFR interpretation is based on the correlation among the parameters, which are known to be highly variable. Second, HCPs should document the patient’s dietary intake and any recent supplement use at the time of sample collection, particularly in non-fasted patients, to aid result interpretation. Given the limited number of laboratories analyzing this parameter, appropriate training should be extended to ensure standardized knowledge of how to measure this parameter.

Serum FGF23 levels should be tested to ascertain the etiology of hypophosphatemia [14]; the assay should be performed only in laboratories with proven expertise. As the results from commercially available assays are notoriously difficult to interpret and not easily comparable, we suggest that FGF23 serum levels be read in the context of other biochemical parameters either involved in its regulation or whose levels are known to oscillate according to fluctuations in the FGF23 level. Specifically, serum FGF23 should not be evaluated in isolation; at a minimum, serum phosphate levels should be measured concurrently to ensure clinically meaningful interpretation.

Once the differential diagnosis indicates a large change of XLH, testing for *PHEX* gene alterations is key for the definite diagnosis of the disease [12, 14], and it ensures eligibility for burosumab reimbursement in Italy and other European countries [101]. *PHEX* gene testing can also serve to rule out the possibility of XLH in patients with suspected TIO [46]. Therefore, genetic testing should be prioritized during the diagnostic workup of the patient despite its elevated cost. As the comprehensive detection of genetic anomalies involves the use of both NGS and MLPA, and results interpretation requires high technical expertise; samples should be sent to and analyzed by specialized laboratories and/or research centers.

In patients with suspected TIO, tumor localization necessitates sophisticated imaging procedures, for which a stepwise approach is necessary to minimize radiation exposure and prevent unnecessary costs. Among functional imaging techniques, ^68^Ga-DOTA-Phe^1^-Tyr^3^-Thr^8^-octreotate positron emission tomography/computed tomography (^68^Ga-DOTATATE PET/CT) and other similar systems exploiting high-affinity binders to SSTRs (^68^Ga-DOTAT-NOC PET/CT and ^64^Cu-DOTATATE-PET/CT) appear to be among the most sensitive and specific techniques for detecting tumors in TIO patients [129–132]. As such, these techniques should be prioritized in cases of suspected TIO, and their accessibility should be broadened. Importantly, imaging should include a whole-body scan, covering both the upper and lower limbs, as routine protocols may exclude these regions and potentially miss tumour localization.

### Suggestions for improved management of XLH patients

XLH patients should be treated as soon as possible after receiving the diagnosis, with the goal of either healing existing rickets or preventing their development, enhancing growth and improving bone deformities [12, 14, 107]. Treatment with burosumab is recommended; if patients are not eligible, they should receive therapy with oral phosphate and active vitamin D. Nevertheless, recent international guidelines also suggest the use of burosumab in infants below 1 year of age (between 6 and 12 months) as the benefits of the treatment are expected to outweigh the undesired effects, despite some uncertainty [25]. Besides, the switch to burosumab should be promptly considered in children and adolescents between 1 and 17 years old who are receiving therapy with oral phosphate and active vitamin D in case of scarce or absent clinical benefits, adverse events and/or lack of adherence [14]. Once treatment is initiated, patients should be strictly followed to monitor the efficacy of the therapy and the development of adverse events. In patients receiving burosumab, efforts should be focused on maximizing its effects, for which dose adjustments may be required based on treatment response [14]. In adults, dosage should be tailored to keep serum phosphate near the lower to the mid-normal range for age at peak and near or just below normal at the trough while strictly preventing any instance of hyperphosphatemia throughout the dosing period [26].

While the updated guidelines state that treatment response should be based on biochemical and clinical improvements, no definitive cut-off values have been established, as these depend on the disease’s severity at treatment initiation [14]. Given the inability to establish standardized cut-off values to quantify the extent of patients’ response to burosumab, we encourage setting treatment goals in a highly personalized manner. Recommendations about tests (such as hearing tests, dental assessments, radiological imaging, and physical performance) and monitoring frequency have been recently updated [14, 25, 26]. It is paramount to implement these guidelines systematically and have standard methodologies in place to ensure a comprehensive evaluation of patients. On the other hand, in addition to adhering to recommendations, we also encourage designing a personalized follow-up strategy, taking into account individualized treatment goals as well as the patient’s age, medical history, clinical and biochemical features, radiological manifestations, and risk of complications. Within the multidisciplinary team, a bone specialist (i.e., orthopedic consultant, rehabilitation physician, rheumatologist) should serve as the key figure in the patient’s routine care.

Of note, children and adolescents should be monitored differently because of the evolving musculoskeletal characteristics during growth; for this reason, tools and patient-reported outcomes employed in adult patients may also be appropriate to track the disease in adolescent patients [101]. Although not widely used, magnetic resonance imaging (MRI) could be a valid instrument for assessing growth plate closure and improvement in bone abnormalities in adolescents and young adults [133–135]. As previously noted, more research on adolescent patients is warranted to better define the best strategies for their care [101].

Once started during childhood, burosumab treatment should ideally be continued in adulthood (i.e., approximately until the middle of the third decade) without interruption to maximize bone mass accrual [14]. As explained above, the presence of fractures or pseudofractures is required in many countries for adults to be eligible for therapy. Therefore, it is of utmost importance that patients over 17 years old with XLH are proactively monitored to diagnose fractures and/or pseudofractures to continue (or resume) burosumab treatment. Multiple-directional X-rays, bone scintigraphy (99mTc-methylene diphosphonate/hydroxymethylene diphosphonate), and T2-weighted fat-suppressed MR images are useful for identifying small pseudofractures [136]. Adults should undergo annual screening for fractures and pseudofractures [26].

In general, the management of patients should be optimized during the transition period between childhood and adulthood. Efforts to foster the development of transition healthcare models are crucial to streamline the continuation of care and avoid patient loss at follow-up. These models should ideally include the establishment of clinics shared between pediatric and adult specialists, although these may not be feasible or readily available. Hence, as a minimum, we advocate close and sustained collaboration where pediatricians can support adult specialists by providing educational resources and developing transition protocols and timelines tailored to each patient’s needs [120, 121]. From the patient’s perspective, raising awareness of the disease among adolescents is essential to promote their empowerment, prepare them for changes in care and smooth the transition process. Detailed information on available treatments and patient support resources should be provided [25, 120]. In this context, the role of patient support groups is essential to ensure education and provide details about specialized care and psychological support [26].

### Suggestions for improved management of patients with TIO

As for XLH, medical treatment should be initiated as soon as possible after TIO has been diagnosed. If the tumor has been localized and surgery is planned, patients should receive therapy with oral phosphate and active vitamin D before surgery, as burosumab is approved only for nonresectable/nonlocalized tumors [20]. After surgery, patients should be regularly monitored to assess their response and detect possible recurrence. As previously suggested [45], serum phosphate levels should be measured every 6 months and then yearly at a minimum. More tailored follow-up should be programmed for patients undergoing medical treatment with either therapy with oral phosphate and active vitamin D or burosumab, in whom surgery cannot be performed, and the tumor is still present.

**Table 1:** Expert recommendations for improved diagnosis and management of X-linked hypophosphatemia and tumor-induced osteomalacia

General advice
Increase networking among all HCPs potentially involved in XLH and TIO diagnosis and management → Hold periodic multidisciplinary meetings to share clinical cases and exemplify best practices → Enhance education, promote networking and raise awareness of the updated recommendations on XLH diagnosis and management → Involve experts in metabolic bone diseases
**Suggestions to improve XLH and TIO diagnosis**
In the presence of clinical signs and symptoms suggestive of XLH, prioritize the following diagnostic tests in this order: 1. Serum phosphate level 2. If hypophosphatemia is detected, measure TmP/GFR 3. Measurement of serum FGF23 levels 4. Analysis of *PHEX* gene mutations a. To confirm XLH in absence of family history of X-linked inheritance b. To exclude XLH and confirm TIO in case of diagnostic doubt 5. (TIO only): prioritize ^68^Ga-DOTATATE PET/CT imaging to localize the tumor
→ Advice to conduct diagnostic tests and interpret results: Extend training to calculate TmP/GFR among laboratories → Consider the values of multiple biochemical parameters to interpret serum FGF23 levels → Enhance collaboration with specialized laboratories and/or research centers to assess the pathogenetic relevance of VUS
**Suggestions to improve XLH management**
Initiate treatment as soon as possible. Burosumab is now the recommended first-line therapy if patients are eligible in children from 1 to 17 years.
Set personalized goals to quantify patient’s clinical improvement
Systematically implement guidelines for standard monitoring of patients while designing a personalized follow-up strategy
Ensure the involvement of bone specialists as key figures in routine care and integral members of the multidisciplinary team
Monitor adolescent patients with the same tools and procedures used for adult patients
Active search for fractures and pseudofractures to ensure burosumab treatment continuation during adulthood with advanced imaging technologies (e.g.,multiple-directional X-rays, bone scintigraphy, T2-weighted fat-suppressed MRI)
Smooth transition of care between childhood and adulthood and avoid loss of patient to follow-up: → Ideally, shared clinics should be established between pediatricians and adult specialists → As a minimum, pediatricians should support adult specialists in the continuity of care process through educational resources/transition protocols → Raise patients’ awareness and empowerment
**Suggestions to improve TIO management**
Initiate treatment as soon as possible → With oral phosphate and vitamin D therapy, if surgery is planned → With burosumab, if the tumor cannot be removed and patients are eligible
After tumor excision, monitor serum phosphate levels every 6–12 months
If the tumor has not been removed and patients are undergoing medical treatment, a tailored follow-up should be programmed

^68^Ga-DOTATATE PET/CT: ^68^Ga-DOTA-Phe^1^-Tyr^3^-Thr^8^-octreotate positron emission tomography/computed tomography; FGF23: fibroblast growth factor 23; HCP: healthcare professional; MRI: magnetic resonance imaging; TIO: tumor-induced osteomalacia; TmP/GFR: tubular maximum reabsorption of phosphate per glomerular filtration rate; XLH: X-linked hypophosphatemia; VUS: variant of uncertain significance

## Conclusion

XLH and TIO are rare but burdensome FGF23-related hyperphosphaturic hypophosphatemia cases whose diagnosis is extremely complex and often delayed. The approval of burosumab represents a turning point for the treatment of these conditions, exceeding the efficacy and safety of therapy with oral phosphate and active vitamin D. Given the multifaceted and ambiguous symptoms and the limited experience of these rare diseases, the management of XLH and TIO patients in clinical practice is far from optimal, resulting in fragmented care and minimal improvements.

Increased awareness among HCPs, facilitated networking among various specialists, and fostering seamless multidisciplinary collaboration is essential to enhancing the care of patients with XLH and TIO. These efforts should culminate in the implementation of standardized diagnostic procedures and follow-up protocols to guarantee the best outcomes for patients.

## Data Availability

Not applicable.
